# Supplementary material for: Prognostic significance of SHP2 (PTPN11) expression in solid tumors: A meta-analysis
Source: PLoS One. 2022 Jan 21;17(1):e0262931. doi: 10.1371/journal.pone.0262931 (PMC8782321; doi:10.1371/journal.pone.0262931)
Supplement: S1 Table — (DOC) [file pone.0262931.s002.doc]

TableS1 Results of quality assessment using the Newcastle-Ottawa scale for included studies

| cohort studies | **Selection** | | | | **Comparability control for important factor** | **Outcome** | | | Quality stars  (NOS) |
| --- | --- | --- | --- | --- | --- | --- | --- | --- | --- |
| **Author(year)** | **Represent ativeness of the exposed**  **cohort** | **Selection of the non exposed**  **cohort** | **Assessment of exposure** | **Demonstration that outcome of interest was not present at start of study** |  | **Assessment of outcome** | **Was follow up long enough for outcomes to occur** | Adequacy of **follow up** of cohorts |  |
| **Chengying Jiang2012** | ★ | ★ | ★ | ★ | ★ | ★ | ★ | ★ | **8** |
| **Jing Jiang2013** | ★ | ★ | ★ | ★ | ★★ | ★ | ★ | ★ | **9** |
| **JIA GU2014** | ★ | ★ |  | ★ | ★ | ★ | ★ | ★ | **7** |
| **Tao Han2015** | ★ | ★ | ★ | ★ | ★ | ★ | ★ | ★ | **8** |
| **Jiawei Zheng2016** | ★ | ★ | ★ | ★ | ★★ | ★ | ★ | ★ | **9** |
| **Chen Qi2017** | ★ | ★ | ★ | ★ |  | ★ | ★ | ★ | **7** |
| **Yan Huang2017** | ★ | ★ | ★ | ★ | ★★ | ★ | ★ | ★ | **9** |
| **Jun Cao 2018** | ★ | ★ | ★ | ★ | ★ | ★ | ★ | ★ | **8** |
| **Min-Kyung Kim2018** | ★ | ★ | ★ | ★ |  | ★ | ★ | ★ | **7** |
| **Niki Karachaliou2019** | ★ | ★ |  | ★ | ★ | ★ | ★ | ★ | **7** |
| **Ivan Macia2020** | ★ | ★ | ★ | ★ |  | ★ | ★ | ★ | 7 |
| **Jing Chen2020** | ★ | ★ | ★ | ★ | ★★ | ★ | ★ | ★ | 9 |
| **Jing Chen2020** | ★ | ★ | ★ | ★ | ★★ | ★ | ★ | ★ | 9 |
| **Jing Chen2020** | ★ | ★ | ★ | ★ | ★★ | ★ | ★ | ★ | 9 |
| **Case control studies** | **Selection** | | | |  | **Exposure** | | |  |
|  | **Adequate definition of cases** | **Represent ativeness of the cases** | **Selection of controls** | **Definition of controls** | **Comparability control for important factor** | Ascertainment of  **exposure** | Same method of Ascertainment for cases and controls | Non response rate |  |
| **Jin Soo Kim 2009** | ★ | ★ | ★ | ★ |  | ★ | ★ | ★ | 7 |
| **L.B. Dong2013** | ★ | ★ | ★ | ★ |  | ★ | ★ | ★ | 7 |
| **ZHONG-QIANHU2015** | ★ | ★ | ★ | ★ |  | ★ | ★ | ★ | 7 |
