# Supplementary material for: Prognostic significance of SHP2 (PTPN11) expression in solid tumors: A meta-analysis
Source: PLoS One. 2022 Jan 21;17(1):e0262931. doi: 10.1371/journal.pone.0262931 (PMC8782321; doi:10.1371/journal.pone.0262931)
Supplement: Attachment — Submitted filename: Plos_comments.docx [file pone.0262931.s003.docx]

The article seems to have a sound methodology and the author explained well with the meta-analysis part to show the prognostic value of SHP2. I suggest the author with minor suggestion to be done below

1.       To check for grammar correction

2.       Author can focus on the explanation part for the data utilized for Subgroup Meta-analyses and publication bias.
