# Supplementary material for: Prognostic significance of SHP2 (PTPN11) expression in solid tumors: A meta-analysis
Source: PLoS One. 2022 Jan 21;17(1):e0262931. doi: 10.1371/journal.pone.0262931 (PMC8782321; doi:10.1371/journal.pone.0262931)
Supplement: Attachment — Submitted filename: Response to Reviewers.doc [file pone.0262931.s004.doc]

1. To check for grammar correction

Response: Grammar correction has been performed.
2. Author can focus on the explanation part for the data utilized for Subgroup Meta-analyses and publication bias.

Response: The explanation for the data has been supplemented.

These studies provided almost all the available evidences worldwide on the role of SHP2 in the prognosis of solid tumors. A advantage of bringing together worldwide evidences on the association between SHP2 and the prognosis of solid tumors was that there were large numbers of cases to assess reliably whether the association varies by tumour subtype. We found that high SHP2 expression was associated with shorter OS in two subtypes of solid tumors of pancreatic carcinoma and laryngeal carcinoma, which had little effect on OS in other solid tumors. We also found a small increase in DFS of colorectal carcinoma in high SHP2 expression compared with low one, rather than other solid tumors. It was more puzzling that the role of SHP2 in the prognosis of hepatocellular carcinoma. High SHP2 expression was associated with shorter DFS while early clinical stage in hepatocellular carcinoma. These further suggested that SHP2 might have more complex mechanisms in hepatocarcinogenesis and play complicated effects on hepatocellular carcinoma.

Egger’s test did not show significant difference in clinical stage, DFS, and OS from the studies. That meant the non-existent of publication bias in clinical stage, DFS and OS, and these results were reliable.
